# Transthoracic Cross-Clamping Versus Endo-Aortic Balloon Occlusion in Minimally Invasive Mitral Valve Surgery: A Single-Center Retrospective Cohort Study

**DOI:** 10.3390/medicina62020370

**Published:** 2026-02-13

**Authors:** Ahmed Shazly, Vincenzo Caruso, Arvind Singh, Alessia Rossi, Inderpaul Birdi, Antonio Bivona

**Affiliations:** 1 Essex Cardiothoracic Centre, Mid and South Essex Foundation Trust, Basildon SS16 5NL, UK; arvind.singh8@nhs.net (A.S.); alessia.rossi@nhs.net (A.R.); antonio.bivona@nhs.net (A.B.); 2 St Thomas’ Hospital, London SE1 7EH, UK; vincenzo.caruso@gstt.nhs.uk; 3 The Keyhole Heart Clinic, London SW5 0TU, UK

**Keywords:** minimally invasive surgery, transthoracic clamp, endo-aortic clamp

## Abstract

*Background and Objectives:* Minimally invasive surgery (MIS) has become a cornerstone approach in cardiac surgery. A debate persists regarding the optimal aortic clamp occlusion strategy, with limited comparative data. The two principal strategies, which are transthoracic cross-clamping (TTCC) and endo-aortic balloon occlusion (EABO), offer distinct advantages, but comparative clinical data remain limited. This study compares the two techniques in terms of procedural safety and early outcome. *Materials and Methods:* This single-center retrospective study included consecutive adult patients undergoing elective MIS via video-assisted right mini-thoracotomy between 2012 and 2018 for mitral valve surgery. Tricuspid repair, atrial fibrillation and redo surgery were included in the final cohort. Aortic occlusion was performed with transthoracic cross-clamping (TTCC) or endo-aortic balloon occlusion (EABO). Primary endpoints were intra-operative complications and the rate of conversion to full sternotomy; secondary outcomes were overall mortality and Society of Thoracic Surgeons (STS)-defined comorbidities. *Results:* A total of 163 patients were analyzed (TTCC: *n* = 99, 60%; EABO: *n* = 64, 40%). While both techniques demonstrated equivalent safety profiles (overall mortality: 0%), EABO was associated with higher conversion to full sternotomy [(*n* = 7, 10.9%) vs. TTCC (*n* = 1, 1.3%), *p* = 0.016]. In a generalized estimation equations (GEE) model, no patient-level covariate predicted conversion, suggesting technical or procedural factors as the primary contributors. In addition, EABO was associated with longer cross-clamp time [median: 87 min (IQR: 73, 100) vs. TTCC median: 77 min (IQR: 65.5, 87.5), *p* = 0.03]. Stroke, acute kidney injury, respiratory failure, reoperation and wound infection did not differ significantly; also, hospital stay was similar between groups. *Conclusions:* In this single-center series, EABO showed longer operative times and a higher conversion rate to sternotomy, but without excess mortality or major complications. This may be correlated with the initial learning phase and redo cases; further comparison is needed to assess the benefits of EABO.

## 1. Introduction

Minimally invasive surgery (MIS) via right mini-thoracotomy is now an established alternative to median sternotomy [1], driven by innovation in imaging and instrumentation, and offering reduced surgical trauma, faster recovery and improved cosmesis in appropriately selected patients [2,3]. A central technical issue is the method of aortic occlusion, which must ensure reliable myocardial protection while preserving procedural safety [4].

Two main strategies are widely used: transthoracic cross-clamping (TTCC), applied directly to the ascending aorta under thoracoscopic guidance, and endo-aortic balloon occlusion (EABO), introduced via the femoral artery and inflated in the ascending aorta.

TTCC offers direct mechanical occlusion and straightforward cardioplegia delivery. It is simple and cost-effective but requires dissection around the ascending aorta, which may be challenging in redo surgery or calcified aortas. EABO enables a “no-touch” aortic strategy that avoids dissection of the ascending aorta and is therefore attractive in redo cases; however, EABO introduces device-specific considerations such as balloon positioning, risk of migration, and vascular access complications that require coordinated imaging and perfusion monitoring [5,6].

Comparative evidence indicates broadly similar early safety profiles for TTCC and EABO, but with differences in operative metrics such as cross-clamp and bypass times, in addition to conversion rates that appear sensitive to institutional experience and case mix [6,7]. Systematic reviews and pooled analyses emphasize that heterogeneity in study design and surgeon experience complicates direct comparisons, and that learning curve effects can increase early operative times and conversion rates for centers newly adopting EABO [5,8,9].

Learning curve and team coordination are therefore critical; contemporary series show that outcomes improve as teams gain experience with balloon positioning, transesophageal echocardiography guidance, and peripheral cannulation protocols, reducing device-related complications and the need for conversion [6,10]. Given these factors, the choice between TTCC and EABO should be individualized based on aortic anatomy, redo status, peripheral vascular access, and institutional expertise [9,11].

The aim of this study is to compare intra-operative metrics and early outcomes of TTCC versus EABO in a consecutive cohort of patients undergoing minimally invasive mitral surgery in a high-volume tertiary center.

## 2. Materials and Methods

### 2.1. Study Design

This retrospective cohort study included adult patients who underwent MIS at a single UK tertiary center between October 2012 and January 2018. Cases were identified from a prospectively maintained database and cross-checked with operative records. Inclusion criteria were patients undergoing elective isolated mitral valve surgery (repair or replacement) or concomitant with tricuspid repair, atrial septal defect closure, and atrial fibrillation ablation via right mini-thoracotomy, with peripheral cardiopulmonary bypass (CPB). Exclusion criteria were urgent/emergency cases, dilated aortic root (>35 mm) and/or ascending aorta (>40 mm), the presence of grade higher than mild of aortic regurgitation and isolated non-mitral procedures. The final cohort was then divided in two groups: TTCC and EABO.

### 2.2. Surgical Technique

All the patients underwent a minimally invasive approach in the form of video-assisted right mini-thoracotomy (generally through a 4 cm incision) in the inter-areolar area or along the anterior axillary line. For all the patients, a peripheral CPB was instituted using a femoral approach via a Seldinger technique and under trans-esophageal echocardiography guidance; when EABO was used, its balloon positioning was also verified by arterial line pressure waveform analysis. For TTCC, a straight Chitwood clamp (Scanlan International, Saint Paul, MN, USA) was always used, while all the cases of EABO were performed using an IntraClude^®^ device (Edwards Lifesciences LCC, Irvine, CA, USA). Myocardial protection was always achieved with antegrade cold blood cardioplegia administered every 20 min directly in the aortic root, using a direct cannula (type DLP^®^, Dual Lumen aortic root cannula (Medtronic, Inc., Minneapolis, MN, USA) with separated vent line) (TTCC group) or through the delivering lumen of the endoballon (EABO group).

### 2.3. Endpoints

Primary endpoints were intra-operative complications (defined as evidence of aortic dissection and device migration), and the rate of conversion to full sternotomy. Secondary outcomes were all-cause operative mortality (defined as in-hospital mortality or 30-day mortality, including patients who died after 30 days but without discharge), Society of Thoracic Surgeons (STS)-defined comorbidities [stroke (persistent (more than 24 h) neurological deficits and confirmation of brain infarction or hemorrhage on imaging), acute kidney injury (decline in renal function with need for dialysis), respiratory failure (reintubation, tracheostomy or need of prolonged ventilation), re-exploration (return to the theater for post-operative bleeding and/or pericardial effusion or cardiac arrest), or deep sternal wound infection (wound infection occurring within 30 days from surgery)], and hospital stay.

### 2.4. Statistical Analysis

Statistical analysis was conducted using the STATA version 19 software package (StataCorp LLC, College Station, TX, USA) [12] and the R studio software package, version 2024 (Posit Software, PBC, Boston, MA, USA) [13]. Continuous data were expressed as means and standard deviations (SD) or medians and interquartile ranges (IQR); categorical variables were reported as counts and percentages. The normality of distribution of the continuous variables was tested by the Shapiro–Wilk test. Data analysis bias was reduced with the following statistical corrections: missing values (missingness identified as completely at random) were replaced using both linear interpolation and multiple imputation; outliers were transformed with logarithmic functions. Cumulative probability of death was estimated by use of the Kaplan–Meier method. Continuous variables were analyzed via the Wilcoxon signed-rank test; categorical variables were assessed with Fisher’s exact test. A generalized estimation equation (GEE) was used to assess predictors for conversion rate. For all tests, a *p*-value < 0.05 was considered statistically significant.

## 3. Results

### 3.1. Patient Characteristics

A total of 163 patients met the inclusion criteria, of whom 98 (60%) underwent aortic occlusion using TTCC and 65 (40%) using EABO. Median age was similar between groups [TTCC: 66.5 years (QR 56, 73) vs. EABO: 67 years (IQR 56, 70), *p* = 0.43].

Female patients accounted for 33% (*n* = 27) of the TTCC group and 41% (*n* = 26) of the EABO group. Redo surgery was more frequent in the EABO group compared with the TTCC group (*n* = 7, 10.7% vs. *n* = 4, 4.0%, *p* = 0.039).

Other baseline pre-operative characteristics are summarized in Table 1.

### 3.2. Operative Details

Operative and immediate post-operative details are summarized in Table 2. In both the groups, the rate of mitral valve repair was high (total *n* = 152, 93.2%; TTCC: *n* = 93, 95% vs. EABO: *n* = 59, 91%), with a smaller proportion undergoing mechanical or bioprosthetic valve replacement (total *n* = 11, 6.8%; TTCC: *n* = 5, 5% vs. EABO: *n* = 6, 9%). Concomitant procedures were atrial fibrillation ablation (*n* = 5, 3%), atrial septal defect closure (*n* = 5, 3%) and tricuspid valve repair (*n* = 4, 2.5%). Aortic cross-clamp time was longer for EABO (median: 87 min, IQR: 73, 100) than for TTCC (median: 77 min, IQR: 65.5, 87.5), *p* = 0.03; however, there were no significance differences in cardiopulmonary bypass (CPB) times [TTCC: 107 min, IQR: 94, 133 vs. EABO: 112 min, IQR: 73–100)], *p* = 0.51.

### 3.3. Early Outcomes

No aortic dissection or device migration was observed in either group (*n* = 0, 0%).

Conversion from MIS to sternotomy occurred more often for EABO (*n* = 7, 10.9%) than for TTCC (*n* = 1, 1.3%), *p* = 0.016. Conversion rate was not related to redo surgery (conversion for redo: *n* = 0, 0%, *p* = 0.98). Sternotomy was necessary when there was an inability to safely pass the guidewire for femoral cannulation (TCC: *n* = 0, 0% vs. EOBA: *n* = 2, 3%), an inability to arrest the heart (TCC: *n* = 0, 0% vs. EABO: *n* = 1, 1.5%), or ventricular fibrillation (VF) (TCC: *n* = 1, 1.3% EABO: *n* = 4, 6.1%). All VFs happened after weaning off CPB and were refractory to external direct current cardioversion (DCCV); for these cases, the decision of sternotomy was taken for haemodynamic instability and presence of intra-cardiac air. EABO patients recovered after internal DCCV and de-airing maneuvers. The one TTCC patient required reinstitution of CPB; trans-oesophageal echocardiogram showed reduced flow to the circumflex artery, restored after re-implantation of mitral annuloplasty device.

In a generalized estimating equation (GEE) logistic model adjusting for age, sex, logistic EuroSCORE and ejection fraction, none of the covariates showed a statistically significant association with conversion from minimally invasive to sternotomy [age: odd ratio (OR) = 1.05, confidence interval (CI) = 0.92–1.19; sex: OR = 0.99, CI = 0.06–7.02; logistic EuroSCORE: OR = 0.18, CI = 0.02–1.15; ejection fraction: OR = 1.99, CI = 0.88–4.49].

In-hospital or 30-day mortality was null for both groups (*n* = 0, 0%).

The incidence of post-operative stroke was similar (EABO: *n* = 1, 1.5%; TTCC: *n* = 1, 1%, *p* = 0.094); similarly, there were no difference in rates of respiratory failure (EABO: *n* = 1, 1.5%; TTCC: *n* = 1, 1%, *p* = 0.094), reoperation (*n* = 0, 0% in all groups), renal failure (*n* = 0, 0% in all groups) or deep wound infection (*n* = 0, 0% in all groups).

No differences were observed in terms of intensive care [EABO: 25.8 h, IQR (24, 65); TTCC: 27 h, IQR (23, 48), *p* = 0.88] or median hospital lengths of stay [EABO: 7 days, IQR (6, 10); TTCC: 7 days, IQR (5, 9.5), *p* = 0.20]. Post-operative outcomes are summarized in Table 3.

## 4. Discussion

In this single-center study, TTCC and EABO provided comparable early outcomes in MIS, with low mortality and acceptable rates of neurological, renal and respiratory complications in both groups. These findings align with those from the Mini-Mitral International Registry reported by Kofler et al. [1], who analyzed 733 propensity-matched pairs and found no significant differences between EABO and TTCC; in their study, EABO was associated with slightly longer bypass and intubation times, but overall safety profiles were equivalent, reinforcing the concept that both occlusion strategies are viable when used within experienced programs.

Our results also align within a recent study by Magouliotis’ group in 2024 [9], whose pooled meta-analysis concluded that both techniques are safe and feasible, with no significant differences in early mortality or cerebrovascular accident. Historically, concerns have been raised regarding the potential for retrograde femoral perfusion to increase the risk of neurological events via the ‘sandblasting’ effect on aortic atheroma. However, our finding of equivalent low stroke rates (1.5% vs. 1.0%) aligns with the multi-center experience reported by Zacharias et al. [14], who observed a major stroke rate of only 0.8% across 10 experienced European centers. 

The meta-analysis by Magouliotis et al. [9] also showed a tendency towards longer CPB and cross-clamp times. In our cohort, we similarly observed prolonged CPB/cross-clamp times in the EABO group but did not detect an excess of stroke or overt aortic complications. Our findings contrast with Balkhy’s group’s findings, who reported shorter cardiopulmonary bypass time for the EABO group vs. the EAC group, and no statistically significant differences in cross-clamp time [15]. However, it is noteworthy that, although not statistically significant, redo cases often requiring more complex repair were more frequent in the EABO group in our series, which may have contributed to the longer operative times. An added important contextual consideration is that, in our center, adoption of EABO coincided with the early market release of the device. Research by Holzhey et al. [16] indicates that while basic proficiency in minimally invasive mitral surgery may be achieved early, a stable plateau for complex endo-aortic clamping techniques often requires a volume of 75 to 125 cases.

In our series, we observed a higher conversion rate in our EABO group (10.9% vs. 1.3%). Conversions were primarily related to haemodynamic instability, refractory ventricular fibrillation, or difficulties in establishing safe peripheral access. Importantly, no conversion resulted in adverse neurological or mortality outcomes. The absence of a statistically significant association between patient-level variables (including age, sex, EuroSCORE, and ejection fraction) and conversion risk in the GEE model suggests that conversion was influenced by procedural and technical factors rather than by baseline patient risk. This pattern contrasts with Kofler et al. [1], where EABO was associated with fewer conversions than TTCC, suggesting that balloon occlusion can be deployed with at least equal procedural stability.

A notable advantage of the EABO platform is its utility in redo surgery, to avoid mediastinal dissection. In our cohort, EABO was more frequently utilized for redo procedures (10.7% vs. 4.0%). This approach is supported by recent findings from Prestipino et al. [7], which confirm that EABO can be safely deployed in redo settings to minimize sternal re-entry injuries. Although technical complexity is higher, the avoidance of aortic manipulation offers a safety margin that justifies the use of EABO in this high-risk subgroup.

While our study focused on the thoracoscopic approach, it is pertinent to contextualize these findings within the broader landscape of MIS, particularly regarding robotic assistance. A recent meta-analysis by De Jesus et al. comparing robotic versus non-robotic thoracoscopic repair has demonstrated that while both techniques share excellent safety profiles, robotic platforms generally incur longer cardiopulmonary bypass and cross-clamp times [11]. This is often attributed to the complexity of docking and instrument exchange. However, robotic cohorts often achieve slightly shorter hospital and intensive care unit stays [8]. The choice between robotic and non-robotic MIS depends on learning curve and team expertise [16]. Furthermore, while robotics offers superior visualization, non-robotic thoracoscopic approaches remain more cost-effective in many settings due to significantly lower procedural expenses [10].

Taken together, our data and the external literature suggest that EABO and TTCC are complementary tools. While TTCC remains a reliable, efficient option when safe aortic exposure is feasible, EABO offers a valuable “no-touch” alternative in redo interventions or hostile mediastinal anatomy. The risk–benefit profiles of both techniques depend on patient anatomy, redo status, vascular access, and team expertise. In this context, our single-center series contributes additional data showing that even during the learning phase, early outcomes can remain acceptable provided that institutional protocols emphasize careful patient selection, intra-operative imaging guidance and readiness to convert when safety demands it. This study has several limitations, primarily its retrospective nature, small sample size, and the fact that the results were taken from a previous early phase of MIS; further data will be analyzed for the most recent MIS including formal assessment of the learning curve and cost-effectiveness.

## 5. Conclusions

In this cohort study, TTCC clamp and EABO yielded similar early clinical outcomes, including mortality, stroke, renal dysfunction, and length of stay. Despite this, EABO tended to have longer operative times and higher conversion rates. EABO, with its limitation in aortic manipulation, may be useful for redo surgery. These findings support a tailored approach in which the aortic occlusion strategy is individualized according to aortic anatomy, redo status, and institutional expertise.

## Figures and Tables

**Table 1:** Pre-operative characteristics of the two investigated groups.

	TTCC (*n* = 98)	EABO (*n* = 65)	*p*-Value
Age (years) [median (IQR)]	66.4 (55.6–73)	67 (56–70)	0.43
Female sex, *n* (%)	27 (33%)	26 (41%)	0.39
Redo surgery, *n* (%)	4 (4.0%)	7 (10.7%)	0.039
Logistic EuroSCORE [mean (SD)]	4.3 (0.44)	3.5 (0.4)	0.41
Rhythm *n* (%)			0.59
Sinus rhythm	24 (67)	46 (72)	
Atrial fibrillation	12 (33)	18 (28)	
Hypertension *n* (%)	10 (53)	21 (62)	0.08
Previous stroke *n* (%)	0 (0)	2 (6)	0.05
COPD *n* (%)	3 (16)	5 (15)	0.92
Ejection fraction [mean (SD)]	52.8 (1.2)	53.3 (1.07)	0.46

TTCC: transthoracic cross-clamping; EABO: endo-aortic balloon occlusion; (IQR): interquartile range; COPD: chronic obstructive pulmonary disease.

**Table 2:** Operative details.

	TTCC	EABO	*p*-Value
Mitral valve procedure			
Mitral valve repair *n* (%) - Leaflets resection - Chordae implant - Annuloplasty ring - Annuloplasty band	93 (95) 14 (82) 8 (47) 12 (63) 6 (32)	59 (91) 23 (92) 16 (67) 16 (67) 11 (35)	0.092
Mitral valve replacement *n* (%)	5 (5)	6 (9)	0.10
Aortic cross-clamp time (min) [median (IQR)]	77 (65.5–87.5)	87 (73–100)	0.03
Cardiopulmonary bypass time (min) [median (IQR)]	107 (94–133)	112 (73–100)	0.51
Conversion to sternotomy *n* (%)	1 (1.3%)	7 (10.9%)	0.016
Concomitant procedure (%) - Atrial fibrillation surgery - Tricuspid valve repair - Atrial septal defect closure	3 (3.1) 2 (2) 2 (2)	2 (3) 2 (3) 3 (4.6)	0.21 - 0.11

Data are presented as *n* (%) or median (IQR).

**Table 3:** Early post-operative outcomes.

	TTCC	EABO	*p*-Value
Bleeding (mls) [median (IQR)]	275 (200, 428)	275 (175, 388)	0.98
Red blood cell transfusion (unit) [mean SD]	0.75 (0.18)	0.96 (0.23)	0.41
Post-operative stroke *n* (%)	1 (1.0%)	1 (1.5%)	0.094
Respiratory failure *n* (%)	1 (1.0%)	1 (1.5%)	0.094
Intensive care unit stay (hours) (min) [median (IQR)]	27 (23–48)	25.8 (24–65)	0.88
Hospital length of stay (days)	7 (5–9.5)	7 (6–10)	0.20

## Data Availability

The data underlying this article will be shared upon reasonable request to the corresponding author. Data are not publicly available due to Information Governance.

## Abbreviations

The following abbreviations are used in this manuscript:

MIS	Minimally Invasive Surgery
TTCC	TransThoracic Cross-Clamping
EABO	Endo-Aortic Balloon Occlusion
CPB	Cardio-Pulmonary Bypass
SD	Standard Deviation
IQR	InterQuartile Range
GEE	Generalized Estimation Equation
DCCV	Direct Current CardioVersion
